# Functional Characterization of Ecdysis Triggering Hormone Receptors (AgETHR-A and AgETHR-B) in the African Malaria Mosquito, *Anopheles gambiae*

**DOI:** 10.3389/fphys.2021.702979

**Published:** 2021-07-06

**Authors:** Vikas Jindal, Yoonseong Park, Donghun Kim

**Affiliations:** ^1^Department of Entomology, Kansas State University, Manhattan, KS, United States; ^2^Department of Entomology, Punjab Agricultural University, Ludhiana, India; ^3^Department of Vector Entomology, Kyungpook National University, Sangju, South Korea

**Keywords:** *Anopheles gambiae*, ecdysis triggering hormone, ecdysis triggering hormone receptors, G protein-coupled receptor, neuropeptides

## Abstract

Insect ecdysis behavior, shedding off the old cuticle, is under the control of specific neuropeptides with the top command by the ecdysis triggering hormone (ETH). We characterized the ETH receptor (ETHR) of the malaria mosquito, *Anopheles gambiae*, by manual annotation of the NCBI gene (AGAP002881) and functional analysis, using a heterologous expression system in a mammalian cell line. The two splicing variants of ETHRs, ecdysis triggering hormone receptors (AgETHR-A and AgETHR-B), a conserved feature among insects, included of four (552 aa) and five exons (635 aa), respectively. The main feature of manual annotation of the receptor was a correction of N-terminal and exon-intron boundaries of an annotated gene (AGAP002881). Interestingly, the functional expression of the receptor in Chinese hamster ovary cells required modification of the transcription initiation site for mammalian Kozak consensus. In the calcium mobilization assay using the heterologous expression of each receptor, AgETHR-B showed a higher sensitivity to AgETH-1 (28 times) and AgETH-2 (320 times) than AgETHR-A. The AgETHRs showed specificity only to the ETH group of peptides but not to other groups carrying the C-termini motifs as PRXamide, such as pyrokinin1/DH and pyrokinin2/PBAN. Ecdysis triggering hormone receptors (AgETHR-B) responded to different ETH variants of other insect species more promiscuously than AgETHR-A.

## Introduction

In insect growth and development, shedding old cuticles in the ecdysis involves an innate behavioral sequence orchestrated by a set of neuropeptides. The top command molecule, ecdysis triggering hormone (ETH) is released from Inka cells (Zitnan et al., 1996) at the initiation of the behavior. The hormonal action of the ETH peptide exerts its function by activating ETH receptors (ETHR) in the central nervous system to coordinate preecdysis and ecdysis behaviors. The *eth* gene encodes two similar peptides, i.e., pre-ecdysis triggering hormone and ETH, in *Manduca sexta* (Zitnan et al., 1999). Similarly, in other insects, the *eth* gene encodes two active peptides, ETH-1 and ETH-2 (Park et al., 1999; Arakane et al., 2008; Dai and Adams, 2009; Roller et al., 2010). In addition to the role of ETH in ecdysis, it also acts as an obligatory allatotropin to promote juvenile hormone production for reproduction (Areiza et al., 2014). In *Aedes aegypti*, ETH acts as an allatotropic regulator of juvenile hormone III. Silencing of ETHRs reduced juvenile synthesis by corpora allata (CA), whereas stimulation of ETH increased the juvenile hormone acid methyltransferase (Areiza et al., 2014). Meiselman et al. (2017) reported that ETH-signaling deficit leads to sharply-reduced JH levels and reduction in ovary size, egg production, and yolk deposition in mature oocytes (Meiselman et al., 2017). ETH is also known to play a critical role in the proper functioning of octopaminergic neurons to control the reproductive tract and ovulation and local interneurons of the antennal lobe for male–male courtship inhibition in *Drosophila melanogaster* (Meiselman et al., 2018; Deshpande et al., 2019).

The receptors of ETH peptides, i.e., ETHR, belong to a superfamily of membrane proteins G protein-coupled receptor. Two functional isoforms of the ETHR gene, i.e., ETHR-A and ETHR-B, were first identified in *D. melanogaster* (Iversen et al., 2002; Park et al., 2003). These ETHR genes have been characterized in *M. sexta*, *A. aegypti*, *Tribolium castaneum*, *Schistocerca gregaria*, *Panonychus citri*, and *Bactrocera dorsalis* (Park et al., 1999; Arakane et al., 2008; Dai and Adams, 2009; Roller et al., 2010; Lenaerts et al., 2017; Shi et al., 2017; Zhu et al., 2019).

Two functionally-distinct ETHR (AeETHR-A and AeETHR-B) were identified in *A. aegypti* (Dai and Adams, 2009). In 2003, the genome of *Anopheles gambiae* was sequenced (Holt et al., 2002) and 276 GPCRs (G protein-coupled receptors) were identified through bioinformatic analyses (Hill et al., 2002). In *B. dorsalis,* both receptors play different biological functions, i.e., ETHR-A regulating the ecdysis process, whereas ETHR-B functioning in reproduction (Shi et al., 2017, 2019).

The sequences for putative AgETHR-A and AgETHR-B produced by *in silico* analysis (Roller et al., 2010) have been available in GenBank and VectorBase. This study characterized the two isoforms of ETHR, AgETHR-A and AgETHR-B, from *A. gambiae*. The receptors were heterologously expressed in Chinese hamster ovary (CHO) cells, and the effects of different peptides on these receptors were investigated through a calcium mobilization assay with the CHO cells. The investigation of chemicals activating AgETHR might provide an insight for the development of novel methods to control malaria mosquitoes.

## Materials and Methods

### Molecular Characterization of the AgETHRs

Two sets of primers (PCR and nested PCR) were designed based on the NCBI GenBank accession number XM_312031.5 and XM_003436230.1 to amplify two *A. gambiae* ETHRs: AgETHR-A and AgETHR-B. The cDNA of *A. gambiae* (pupae), obtained from Dr. Maureen Gorman (Kansas State University), was used as a template for the first round of PCR using GoTaq^R^DNA Polymerase (Promega). The PCR was conducted in 20 μl volume, including 5X Go taq colorless buffer, 0.1 μm of dNTPs, and 0.4 μm of each primer (Table 1). The PCR condition was as follows: 95°C for 2 min, then touch down PCR for 10 cycles: 95°C for 30 s, 53°C for 60 s, decreasing of 0.5°C for 9 cycles, 72°C for 1.5 min followed by 26 cycles of 95°C for 30 s, 48°C for 60 s, 72°C for 1.5 min, and final extension of 5 min at 72°C. The diluted PCR product (1:10) obtained in the first PCR round was used as a template for the nested PCR in a total reaction of 20 μl. The PCR condition was as follows: 95°C for 2 min, 34 cycles of 95°C for 30 s, 52°C for 60 s, 72°C for 1.5 min, and final extension of 5 min at 72°C.The PCR products were purified using Zymo PCR Cleanup kit (Zymo Research) and cloned in pGEM^®^-T Easy Vector (Promega) following the manufacturer’s protocol. The positive clones were identified through PCR with nested primers and restriction with *EcoR*I. The nucleotide sequences for clones were determined through the custom services of the Genewiz company. The clones were sequenced in both directions (5' and 3' ends) using T7 and SP6 primers. The nucleotides sequences for AgETHR-A and AgETHR-B were edited in a Sequencher software for determining the variants, which were compared with NCBI GenBank sequences. Seven transmembrane segments of both AgETHR-A and AgETHR-B were identified by TMHMM.^1^

**Table 1:** Details of primers used in the study.

		AgETHR-A	AgETHR-B
PCR	Forward	5'-CCGCTGGCTTTAACGATTC-3'	5'-CCGCTGGCTTTAACGATTC-3'
	Reverse	5'-CTTTCGCTTCCAATCGAACACG-3'	5'-CTCCTCCGTAAACCAGTGC-3'
Nested PCR	Forward	5'-C**GCCACC**ATGCCCCAAATTCCGAAGT-3'	5'-CCGCCAGTGTGTCTGGAATTC**GCCACC**ATGCCCCAAATTCCGAAGT-3'
	Reverse	5'-CATCGGCTGGAATTGTCAAAC-3'	5'-TGCTCGAGCGGCCGCGAATTC-3'

The bold sequences in primers are Kozak sequences added to the primers.

### Phylogenetic Analysis

The protein sequences for ETHR-A and ETHR-B available in NCBI GenBank for *A. aegypti* (ABI93273.1 and ABI93274.1), *D. melanogaster* (NP_650960.2 and NP_996255.1), *T. castaneum* (NP_001076792.1 and NP_001076793.1), *B. mori* (NP_001127741.1 and NP_001165737.1), and AgETHR-A and AgETHR-B from *A. gambiae*, which were sequenced in this study, were used for phylogenetic analysis. The sequences were aligned using CluswalW, and the phylogenetic tree was constructed using the neighbor-joining method, available in the MEGA7 software, with 1,000 bootstrap replicates. The Poisson correction method was used to compute the evolutionary distances, which were in the units of the number of amino acid substitutions per site. All ambiguous positions were removed for each sequence pair (pairwise deletion option). Neuromedin U receptor of *Homo sapiens* (AAG24793.1) was used as the outgroup.

### Synthetic Insect Neuropeptides

We tested different synthetic insect neuropeptides with PRXamide, including four groups: ETH, pyrokinin1/DH, pyrokinin2/PBAN, and CAPA. The following synthetic peptides were utilized to investigate selectivity and sensitivity of each AgETHR-A and AgETHR-B; AgETH1 (SESPGFIKLSKSVPRIa, NEOBiolab, United States), AgETH2 (GDLENFFLKQSKSVPRIa, GenScript United States Inc.), TcETH1 (ENYVLKAAKNVPRIa), TcETH2 (FFMKASKSVPRIa), TcPK1-1 (PGANSGGMWFGPRLa, GenScript United States Inc.), TcPK1-2 (TPHESSVPNERNDDSKETYFWFGPRLa), TcCAPA-1(EPKEPKRNKLASVYALTPSLRVa, PepMicCo. Ltd., Suzhou, China), TcCAPA-2 (RIGKMVSFPRIa); MsPBAN (TRTRYFSPRLa), Pyrokinin2-1 (HVVNFTPRLa, Biomatik, United States), DmETH1 (DDSSPGFFLKITKNVPRLa) and DmETH2 (GENFAIKNLKTIPRIa).

### Functional Assay of the AgETH Receptors

The functional analysis of the AgETH receptors was performed based on the previous description with modifications (Park et al., 2003; Kim et al., 2014; Jiang et al., 2016). The expression vector pcDNA 3.1 (+) (Invitrogen, Carlsbad, CA, United States), including the open reading frame (ORF) for each AgETHR-A and AgETHR-B, was transiently transfected into the CHO cells with a codon-optimized human apoaequorin-containing plasmid (Vernon and Printen, 2002) by using TransIT^®^-2020 (Mirus, Madison, WI, United States). Interestingly, the initial trial for the functional expression with the AgETHR-A and AgETHR-B was unsuccessful. The Kozak sequence for the mammalian Kozak consensus sequence ‘GCCACC’ at the 5' ends of each ORF for each AgETHR-A and AgETHR-B was mutated, allowing for successful functional expression. After approximately 24–28 h, transfected cells were harvested and prepared for the calcium mobilization assay by incubating in assay media DMEM/F12, including coelenterazine h (final concentration 5 μm; AAT Bioquest, Sunnyvale, CA, United States). Different concentrations of synthetic neuropeptides, including AgETH1, AgETH2, and ETH-related neuropeptides (PRXamides), were prepared in a 96-well plate, upon which the transfected cells (~15,000 cells in 50 μl) were applied. The value of luminescence, the indicator or calcium mobilization, was integrated over time for 20 s and normalized to the treatment of either 0.1 or 1 μm of each AgETH1 and AgETH2 positive control response and negative control background values.

## Results

### Molecular Characterization of AgETH Receptors

**Figure 1:**
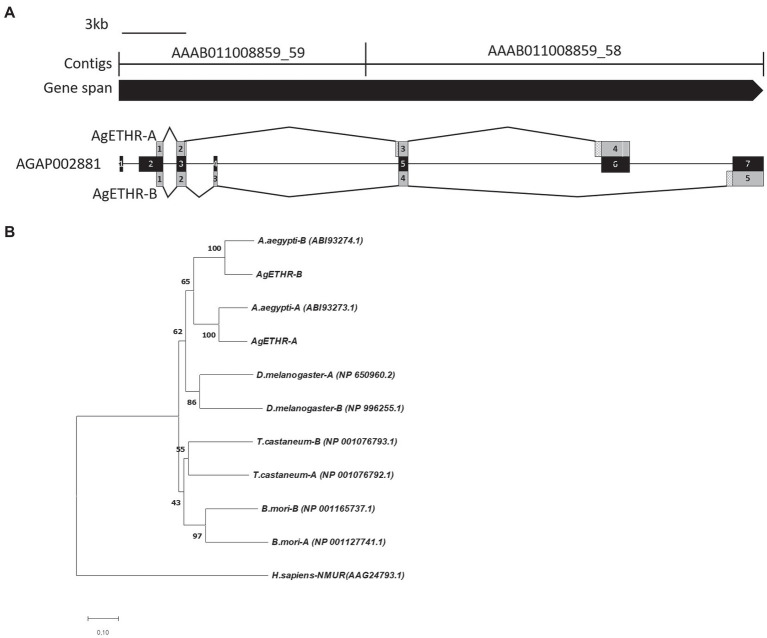
Gene structure and phylogenetic relationship of the AgETH receptors. **(A)** Gene structure of ecdysis triggering hormone receptors (AgETHR-A and AgETHR-B). Empty boxes in exons of AgETHR-A indicated 4 bp and 3 bp fragment deletion. Checkered boxes before exon 3 and 4 of AgETHR-A and exon 5 of AgETHR-B indicate the 5 bp, 51 bp, and 84 bp fragment insertion. **(B)** Phylogenetic relationship of ETH receptors from *Anopheles gambiae, Aedes aegypti, Drosophila melanogaster, Tribolium castaneum,* and *Bombyx mori*. Neuromedin U receptor of *Homo sapiens* was used as an outgroup. The tree was constructed using the Neighbor-Joining method using MEGA7. The percentage of replicate trees in which the associated taxa clustered together in the bootstrap test (1,000 replicates) is shown next to the branches. The evolutionary distances were computed using the Poisson correction method. All positions containing gaps and missing data were eliminated.

The gene structures for AgETHR splicing variants in *A. gambiae* are shown in Figure 1. The total gene spans >34.7 kb, which consisted of two contigs AAAB01008859_59 (13,937 bp) and AAAB01008859_58 (13,289 bp in AgETHR-A and 20,779 bp in AgETHR-B; Figure 1).

The *A. gambiae* AGAP002881 gene includes seven exons, which are alternatively transcribed to produce two variants of the ETHR. However, neither AgETHR-A nor AgETHR-B included the first 675 nt of AGAP002881 (exon 1 and sequences upstream of exon 2). The primers based on the upstream portion of exon 1 from AGAP002881 did not amplify the N-terminal (the first 675 nt) in repeated trials. While the correct N-terminal of AgETHRs was amplified by the primer (Table 1) designed from the upstream of the correct translation initiation site, it was modified by a manual annotation (Figure 1A).

The AgETHR-A gene was comprised of four exons (1,659 nt) encoding 552 amino acids, which was modified by the manual annotation (Figure 1A). The nucleotide sequence of AgETHR-A showed differences at 72 nucleotide positions compared with the sequence of AGAP002881. It included nine nucleotide replacements, 7 nt deletions, and fragments of 5 nt and 51 nt insertion, respectively. The insertion occurred upstream of exon 5 and exon 6 of AGAP002881, which were earlier described as the part of the intron. The exon 4 was the largest in AgETHR-A, which constitutes 74% of the full gene length.

**Figure 2:**
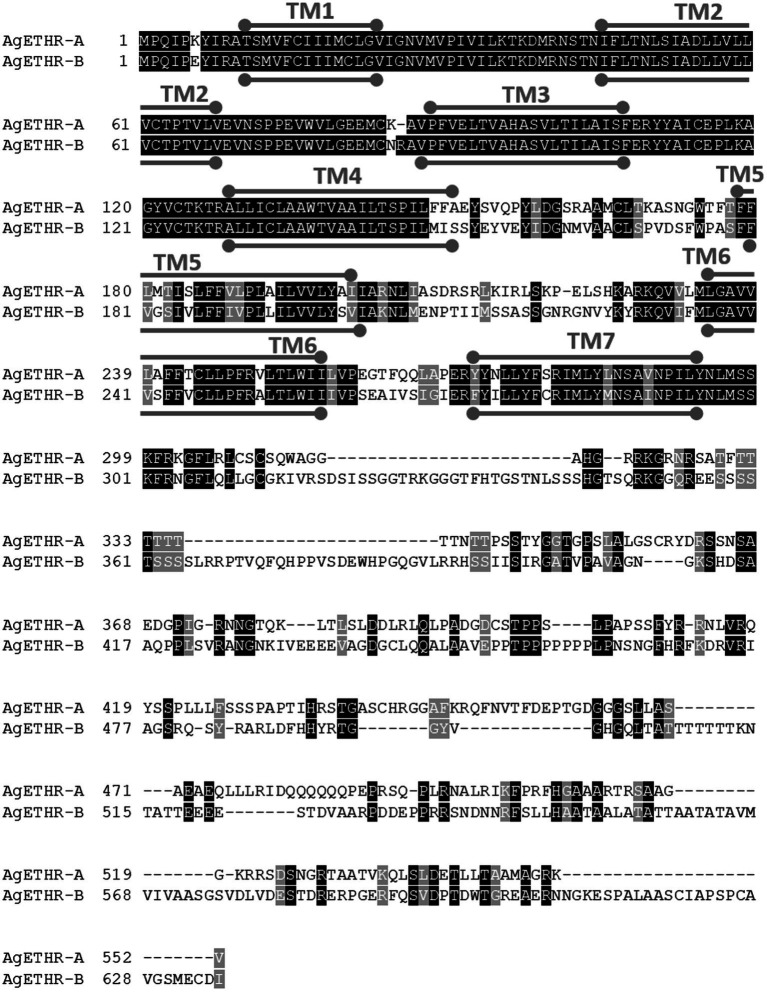
Sequence alignment of AgETHR-A and AgETHR-B. Transmembrane (TM) domains 1–7 are marked either above or below the amino acid sequence of each receptor as a stroked goalpost in gray. The conserved and homology sequence was highlighted in black and gray, respectively.

The alternate splicing variant AgETHR-B encoded 635 amino acids (1908 nt) shared exon 1 and parts of two exons (exon 3 and 5 of AGAP002881) of AgETHR-A, while predicted exons 4 and 7 of AGAP002881 were utilized only for AgETHR-B (Figure 1). Similar to AgETHR-A, the AgETHR-B gene was modified by the manual annotation, including the insertion of 84 nt at the upstream of exon 7 of AGAP002881, where it was predicted as the region of an intron in the genome of *A. gambiae*. Exon 5 was the largest and covered 77% of the full AgETHR-B gene length. In addition, six nucleotides were replaced by other nucleotides in exon 5 of AgETHR-B. The alignment of AgETHR-A and AgETHR-B showed 61.5% homology in the nucleotide sequence. However, there was 43.4% identity and 52.5% similarity in protein sequence among two splicing variants (Figure 2). The manually curated sequences are deposited in GenBank with the accession numbers (MZ027156 and MZ027157).

The phylogenetic analysis led to two distinct clades of ETHR among different insects (Figure 1B). The clade1 grouped ETHR-A and ETHR-B of dipteran insects, i.e., *A. aegypti, D. melanogaster,* and *A. gambiae.* Among these, the mosquito receptors (ETHR-A and ETHR-B) were grouped. ETHR-A for both *A. aegypti* and *A. gambiae* were clustered into a monophyletic group. Similarly, ETHR-B was also clustered into a monophyletic group for both mosquito species. Clade 2 consisted of coleopteran (*T. castaneum*) and lepidopteran (*B. mori*) representatives. All ETHR from insects were placed together and separated from the outgroup Neuromedin U receptor of *H. sapiens*.

### Selectivity and Sensitivity of the AgETH Receptors to a Different Group of Peptides

**Figure 3:**
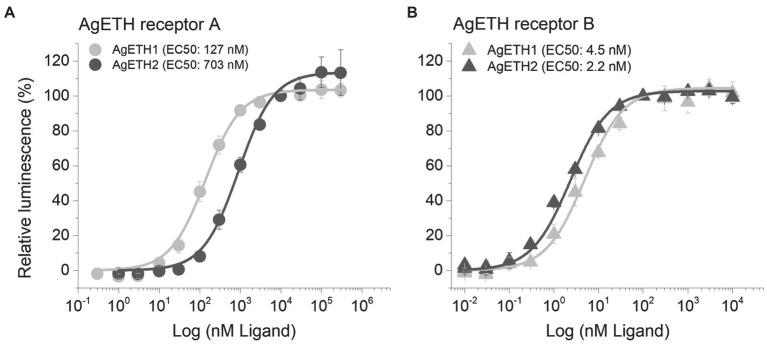
Dose–response relationship to AgETH1 and AgETH2 peptides in heterologously expressed AgETHR-A **(A)** and AgETHR-B **(B)** CHO-WTA11 cell lines. Gray and dark gray represent the response of AgETHR-A and AgETHR-B to synthetic AgETH1 and AgETH2 (from 1 × 10^−2^ to 3 × 10^5^ nm), respectively. EC50 values of each peptide are given in parenthesis. The bars represent the standard error for at least three replicates.

Two subtypes of *A. gambiae* ETHRs (AgETHR-A and AgETHR-B) actively responded and showed dose-dependent responses to AgETH1 and AgETH2, respectively. Overall, AgETHR-B exhibited higher sensitivities than AgETHR-A to both AgETH1 (EC_50_: 4.5 nm vs. 127 nm) and AgETH2 (EC_50_: 2.2 nm vs. 703 nm), respectively (Figure 3). Ecdysis triggering hormone receptors (AgETHR-A) was 4.5 times more sensitive to AgETH1 (EC_50_: 127 nm) than to AgETH2 (EC_50_: 703 nm; Figure 3A). Ecdysis triggering hormone receptors (AgETHR-A) was not activated lower than 3 nm AgETH1 and 10 nm AgETH2 and had maximum response at the level over 10 um AgETH1 and 100 um AgETH2. Ecdysis triggering hormone receptors (AgETHR-B) was one time more sensitive to AgETH2 (EC_50_: 2.2 nm) than to AgETH1 (EC_50_: 4.5 nm; Figure 3B). Ecdysis triggering hormone receptors (AgETHR-B) was not activated lower than 0.1 nm AgETH1 and 0.03 nm AgETH2 and had maximum response at the level over 100 nm AgETH1 and AgETH2.

**Figure 4:**
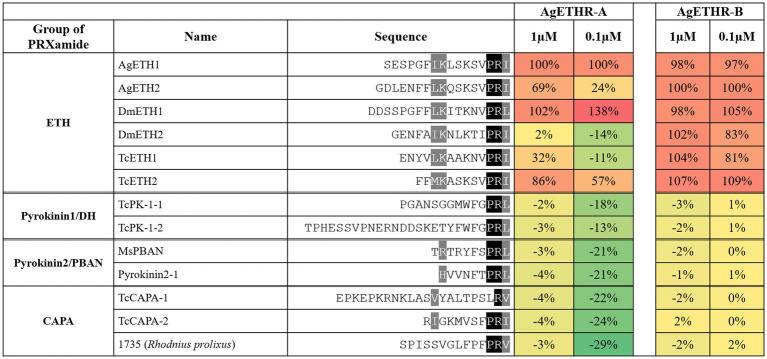
Agonistic activities of the group of PRXamide on AgETHR-A and AgETHR-B. Two concentrations of each neuropeptide were tested on both AgETHR-A and AgETHR-B. The value was normalized by the activity of AgETH1 for AgETHR-A and AgETH2 for AgETHR-B, respectively. The conserved sequences were highlighted in black and gray.

In Addition to AgETH1 and AgETH2, AgETHR-A and AgETHR-B also responded to other insects ETHs (0.1 and 1 μm), including those from *D. melanogaster* (DmETH1 and DmETH2) and *T. castaneum* ETHs (TcETH1 and TcETH2; Figure 4). The response of AgETHR-A and AgETHR-B to other insect neuropeptides was normalized by the reactions to AgETH1 and AgETH2, respectively (Figure 4). Ecdysis triggering hormone receptors (AgETHR-A) responded to DmETH1 and TcETH2 at the level of 0.1 and 1 μm, with small or no response to DmETH2 and TcETH1. Ecdysis triggering hormone receptors (AgETHR-B) responded to all tested insect ETHs, although it showed relatively lower responses to DmETH2 and TcETH1 than other ETHs at the level of 0.1 μm. However, AgETH receptors did not react to ETH-related insect neuropeptides, such as Pyrokinin1/DH, Pyrokinin2/PBAN, and CAPA, which are insect neuropeptides (PRXamide) structurally similar to ETH (Figure 4).

## Discussion

In insects, ETH is the top command molecule neuropeptide and crucial in regulating the ecdysis process (Zitnan et al., 1996; Diao et al., 2016). This neuropeptide hormone activates ETHR and induces internal signal transduction pathways. Two isoforms of ETHR were reported in other insect species (Zitnan et al., 2002; Kim et al., 2006; Dai and Adams, 2009; Roller et al., 2010; Shi et al., 2017). However, only one ETHR was reported in mites and locust (Veenstra et al., 2012; Lenaerts et al., 2017; Zhu et al., 2019).

The NCBI gene prediction of AGAP002881 included seven exons, which were supposed to express AgETHR-A and AgETHR-B by alternative splicing. The primers based on the NCBI gene prediction of AGAP002881 did not amplify the N-terminal of each receptor in repeated trials. In addition, the predicted N-terminal of AGAP002881 was not conserved compared with other ETHRs of mosquito and dipteran insects. However, the primer designed from the second AGAP002881 exon amplified the true N-terminal and translation-initiation site, which were corrected (Figure 1). In addition, the AGAP002881 exon-intron boundaries were corrected by manual annotation based on cloning and sequencing analysis.

The phylogenetic analysis clustered both ETHR-A and ETHR-B of mosquitoes into separate groups, indicating that the subtypes of ETHR from mosquitoes are closely related. The two isoforms might have diverged during the evolution of insects and are supposed to have different roles in insects like ecdysis and reproduction (Shi et al., 2017, 2019). Isoform A of insect ETHR seems likely an ancestral form of ETHR, based on the phylogenetic analysis with alternative exons from holometabolous insects, such as Coleoptera, Lepidoptera, and Diptera, and single exons from Hemiptera, Orthoptera, and Acari (Supplementary Figure S1).

The functional assay of AgETHR-A and AgETHR-B indicated that both were sensitive to AgETH-1 and AgETH-2, but AgETHR-B had higher sensitivity to AgETH-1 (28 times) and AgETH-2 (320 times) than AgETHR-A. A similar activity of ETHR-A and ETHR-B was reported from other insects including *D. melanogaster, B. dorsalis, and M. sexta* (Iversen et al., 2002; Park et al., 2003; Kim et al., 2006; Shi et al., 2017). In contrast, the ETHR-A of *A. aegypti* had higher sensitivity to AeaETH-1 than ETHR-B (Dai and Adams, 2009). The response of ETHRs of *T. castaneum* and *S. gregaris* was found to show similar responses to ETH-1 and ETH-2 (Jiang et al., 2015; Lenaerts et al., 2017).

Ecdysis triggering hormone receptors (AgETHRs) were also tested for their sensitivity for the ETH from *D. melanogaster* and *T. castaneum* and other peptides of PRXamide like CAPA and pyrokinin1/DH and pyrokinin2/PBAN of different insects. Ecdysis triggering hormone receptors (AgETHR-A and AgETHR-B) showed varied sensitivity to all these peptides. Ecdysis triggering hormone receptors (AgETHR-A) responded to DmETH-1 and TcETH-2, while AgETHR-B responded to all tested ETH peptides at a lower concentration. No other peptides belonging to CAPA, pyrokinin1/DH, and the pyrokinin2/PBAN group activated any AgETHRs, confirming their specificity toward ETH peptides only. The ETHR (CG5911b) from *D. melanogaster* was more sensitive to MasETH and MasPETH of *M. sexta* than CG5911a (Park et al., 2003). Similarly, *A. aegypti* ETHRs were activated by DmETH and MasETH but not by MasPETH (Dai and Adams, 2009). Jiang et al. tested a different group of peptides, such as pyrokinin1/DH, pyrokinin2/PBAN, CAPA, ETH, and synthetic peptides, on other receptors, including TcETHR-B of *T. castaneum* (Jiang et al., 2015). Out of 70 peptides, only peptide no. 1,490 from *D. melanogater* showed an 83% agonist activity of TcETHR-B in addition to TcETH-1 and TcETH-2. These studies support our results that ETHR is only sensitive to the ETH group of peptides. Alternate splicing variants of ETHR might be involved in a different physiological role in insects, like in *B. dorsalis*, where ETHR-A regulated the ecdysis process, whereas ETHR-B was important in the reproduction of fruit flies (Shi et al., 2017, 2019).

Overall, we characterized the two isoforms of ETHR in *A. gambiae* and analyzed the response of ETHRs to various PRXamide peptides by using heterologous expression system. Importantly, modification of the transcription initiation site for vertebrate Kozak consensus sequence was required to express the AgETH receptors in CHO cells. The findings will provide the foundation for future studies into the molecular function of ETHRs in various physiological events of mosquitoes and for functional analysis of neuropeptide receptors in other insects.

## Data Availability Statement

The raw data supporting the conclusions of this article will be made available by the authors, without undue reservation.

## Author Contributions

VJ and DK conceived and designed the experiments, performed the experiments, analyzed the data, wrote the paper, prepared figures and/or tables, and reviewed drafts of the paper. YP conceived and designed the experiments, analyzed the data, contributed reagents, materials, and analysis tools, wrote the paper, prepared figures and/or tables, and reviewed drafts of the paper. All authors contributed to the article and approved the submitted version.

### Conflict of Interest

The authors declare that the research was conducted in the absence of any commercial or financial relationships that could be construed as a potential conflict of interest.

## Footnotes

^1^http://www.cbs.dtu.dk/services/TMHMM/

## Supplementary Material

Supplementary Figure S1 Phylogenetic relationship of alternative exons of holometabolous insects. Neuromedin U receptor of *Homo sapiens* was used as an outgroup. The tree was constructed using the Neighbor-Joining method using MEGA7. The percentage of replicate trees in which the associated taxa clustered together in the bootstrap test (1,000 replicates) are shown next to the branches. The evolutionary distances were computed using the Poisson correction method. All positions containing gaps and missing data were eliminated.
