# Supplementary figures and images for: Functional Characterization of Ecdysis Triggering Hormone Receptors (AgETHR-A and AgETHR-B) in the African Malaria Mosquito, Anopheles gambiae
Source: Front Physiol. 2021 Jul 6;12:702979. doi: 10.3389/fphys.2021.702979 (PMC8291126; doi:10.3389/fphys.2021.702979)

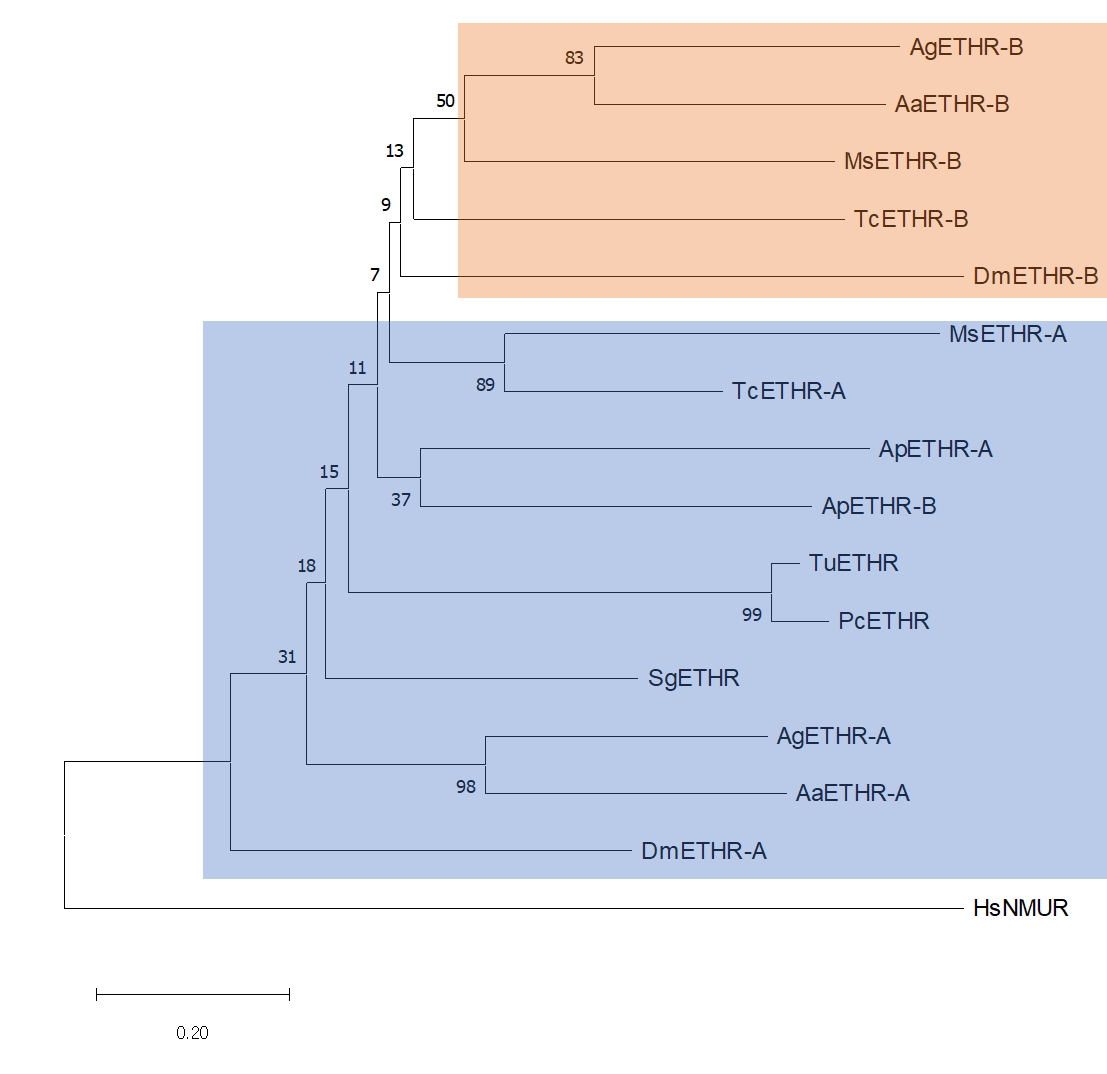

Supplement: Supplementary Figure S1 — Phylogenetic relationship of alternative exons of holometabolous insects. Neuromedin U receptor of Homo sapiens was used as an outgroup. The tree was constructed using the Neighbor-Joining method using MEGA7. The percentage of replicate trees in which the associated taxa clustered together in the bootstrap test (1,000 replicates) are shown next to the branches. The evolutionary distances were computed using the Poisson correction method. All positions containing gaps and missing data were eliminated. [file Image_1.TIF]
